# First Case Report of Mycotic Abdominal Aortic Aneurysm Caused by *Campylobacter fetus* in Serbia

**DOI:** 10.3390/pathogens13090805

**Published:** 2024-09-17

**Authors:** Deana Medic, Milica Devrnja, Nikola Batinic, Djordje Milosevic, Aleksandra Colovic Popadic, Vera Gusman

**Affiliations:** 1 Department of Microbiology with Parasitology and Immunology, Faculty of Medicine, University of Novi Sad, Hajduk Veljkova 3, 21000 Novi Sad, Serbia; deana.medic@mf.uns.ac.rs (D.M.); vera.gusman@mf.uns.ac.rs (V.G.); 2 Institute of Public Health of Vojvodina, Center for Microbiology, 21000 Novi Sad, Serbia; sandra.colovic93@hotmail.com; 3 Department of Surgery, Faculty of Medicine, University of Novi Sad, Hajduk Veljkova 3, 21000 Novi Sad, Serbia; nikola.batinic@mf.uns.ac.rs; 4 Vascular and Endovascular Surgery Clinic, University Clinical Center of Vojvodina, 21000 Novi Sad, Serbia; drmdjole@gmail.com; 5 Institute of Public Health of Vojvodina, Center for Hygiene and Human Ecology, 21000 Novi Sad, Serbia

**Keywords:** *Campylobacter fetus*, mycotic abdominal aortic aneurysm, meropenem, infection

## Abstract

Background: Due to its distinct vascular tropism, *Campylobacter fetus* is recognized as a significant cause of severe systemic infections, especially in immunocompromised individuals, while it is rarely reported as a cause of gastrointestinal infections. Methods: A rare case of mycotic abdominal aortic aneurysm associated with *Campylobacter fetus* detected on the aneurysm wall itself was described. Results: A 68-year-old male was admitted to the hospital due to severe abdominal pain. The patient was afebrile, hemodynamically stable with elevated C-reactive protein levels. A physical examination revealed a palpable, pulsatile, tender mass located in the periumbilical region. Ultrasonography and multi-slice computer tomography angiography (MSCTA) identified an infrarenal abdominal aortic aneurysm with a maximum diameter of 6.5 cm, showing suspicious signs of dissection. Aneurysmectomy with Dacron tube graft interposition was performed. Although the blood cultures remained negative, the culture of the aneurysmal wall grew *Campylobacter fetus*, enabling early diagnosis and targeted antibiotic therapy. The patient was treated with meropenem for two weeks, followed by amoxicillin-clavulanate for another two weeks after hospital discharge. Conclusions: *Campylobacter fetus* associated with abdominal aortic aneurysms represents a life-threatening condition, posing a significant challenge in vascular surgery. Due to the lack of clear guidelines on antibiotic susceptibility testing and the treatment of infections associated with this pathogen, enhanced surveillance of *Campylobacter fetus* is necessary in both human and veterinary medicine.

## 1. Introduction

Representatives of the *Campylobacter* genus are recognized as significant causative agents of bacterial gastrointestinal infections in humans [1]. Infections caused by these Gram-negative bacilli occur most commonly through the consumption of undercooked meat and unpasteurized milk and less commonly through direct contact with domestic animals [2,3]. Over 90% of cases of *Campylobacteriosis* are caused by *Campylobacter jejuni* (*C. jejuni*) and *Campylobacter coli* (*C. coli*). Infections caused by these pathogens typically manifest as mild diarrhea, with systemic spread occurring in rare cases [2]. Unlike the previously described species, *Campylobacter fetus* (*C. fetus*) is a significantly rarer causative agent of infections in humans. *C. fetus* is traditionally recognized as a significant pathogen of livestock. Three subspecies of *C. fetus* have been described: *C. fetus* subsp. *fetus*, *C. fetus* subsp. *venerealis*, and *C. fetus* subsp. *testudinum* [4]. *C. fetus* subsp. *fetus* has been isolated from the intestinal tracts of sheep and cattle, while *C. fetus* subsp. *venerealis is* restricted to cattle. The subspecies show distinct niche preferences: the *C. fetus* ssp. *venerealis* colonizes the genital tract, while *C. fetus* subsp. *fetus* is largely confined to the gut. Both subspecies are a major cause of abortion and infertility, causing substantial losses in bovine, ovine, and caprine herds worldwide [5]. *C. fetus* subsp. *testudinum* was recently described as a novel *C. fetus* subspecies. It has been isolated from reptiles and humans. More detailed genetic studies have indicated a larger genetic distance between mammal- and reptile-associated *C. fetus* subspecies than within mammal-associated *C. fetus* subspecies [6]. The majority of infections in humans are caused by *C. fetus* subsp. *fetus* [5]. In humans, this microorganism is more commonly associated with extraintestinal infections such as bacteremia and meningitis [7]. Infections caused by *C. fetus* primarily occur more frequently in immunocompromised conditions such as malignancies, diabetes mellitus, immunosuppressive therapy, radiotherapy, and similar conditions [8]. Although infections caused by *C. fetus* are not common, mortality due to such invasive infections in certain studies reaches 14% [7]. Extraintestinal infections can be accompanied by secondary localization, leading to the development of infective endocarditis, mycotic aortic aneurysms, meningitis, and similar conditions [9].

Mycotic aneurysms are rare, life-threatening conditions [10,11], representing one of the leading challenges in vascular surgery, and the most common causative agents of such conditions include *Salmonella* spp., *Staphylococcus* spp., and *Streptococcus* spp. [10,11,12,13,14,15]. In addition to the aforementioned pathogens, *C. fetus* has emerged as a significant cause of mycotic aortic aneurysms in recent decades. These conditions arise due to the significant affinity of this microorganism to the vascular endothelium and the high risk of bacteremia [8]. However, due to the challenges in its diagnosis, it is often not identified. Therefore, the use of rapid, sensitive, and specific modern diagnostic methods, such as matrix-assisted laser desorption/ionization time-of-flight mass spectrometry (MALDI TOF), polymerase chain reaction (PCR), pulsed-field gel electrophoresis (PFGE), multiloci sequence typing (MLST), and whole-genome sequencing (WGS), is recommended [16]. This is the first case report of a patient in Serbia with a mycotic abdominal aortic aneurysm (AAA) positive for *C. fetus* on the aneurysm wall.

## 2. Case Report

A 68-year-old patient developed severe abdominal pain without an apparent cause and was admitted to the Emergency Center of the University Clinical Center of Vojvodina the following day due to worsening symptoms. The pain was described as intense, intermittent, localized without radiation and without associated symptoms such as nausea, vomiting or diarrhea. The patient denied any recent gastrointestinal symptoms or fever over the previous weeks. Additionally, there was no recent travel history or contact with animals reported. The patient stated that he lived with his family and consumed only thoroughly cooked meat and pasteurized milk. His past medical history included hypertension and previous hospitalizations for cholecystectomy, inguinal hernia repair, and cataract surgery. There was no history of malignancy, liver disease, AIDS, or similar conditions reported. There were no data on previously received fluoroquinolone therapy, which might be associated with an increased risk of AAA [10]. The only predisposing factor for immunodeficiency obtained from his medical history was long-term alcohol consumption.

**Figure 1:**
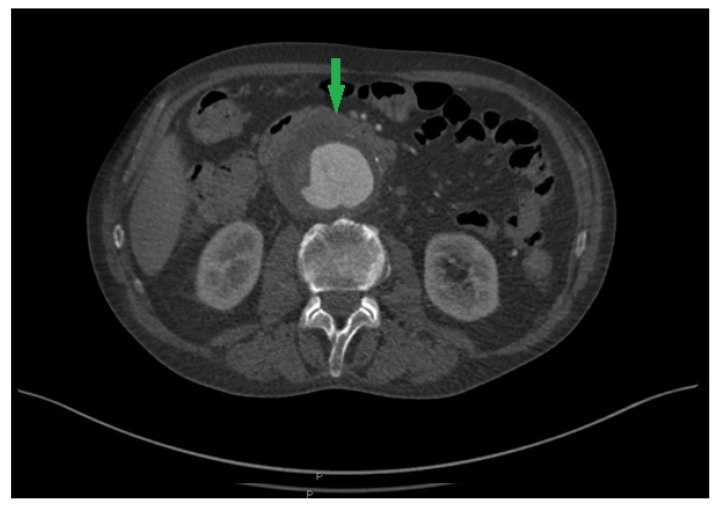
Computed tomography angiography showing an abdominal aortic aneurysm and a deposition of a high-density area of soft tissue on the front wall of the aorta (green arrow).

Upon admission to the University Clinical Center of Vojvodina, the patient was conscious, afebrile, and hemodynamically stable. Laboratory tests revealed significant leukocytosis (15.03 × 10^9^/L) with neutrophil predominance in the differential white blood cell count (84.6%) and elevated C-reactive protein levels (173.2 mg/L). Procalcitonin was measured three days in a row: on the first, second, and third day after the operation, the values were 0.72, 0.61, and 0.36, respectively (reference range: 0.15–0.50%). Fibrinogen was also measured postoperatively, on the first and third day, and the values were 5.75 and 5.98 (reference range: 1.70–4.50 g/L). Platelets were elevated preoperatively (430 × 10^9^/L), but after the operation, they were within the reference range (306 × 10^9^/L). Other hematological and biochemical parameters were within the normal limits. The hemostasis parameters on admission and postoperatively were regularly monitored and were within reference values (aPTT(R) 0.88R, PT(R) 0.95R, PT(INR) 0.94INR). A physical examination revealed a palpable, pulsating mass located in the periumbilical region, tender to palpitation. An abdominal ultrasound identified an infrarenal abdominal aortic aneurysm with a maximal anteroposterior diameter of 58 mm and suspected signs of dissection. Multi-slice computer tomography angiography (MSCTA) confirmed the presence of an infrarenal abdominal aortic aneurysm with a maximum diameter of 6.5 cm, exhibiting linear hypodensity within the lumen suggestive of an intimal flap consistent with dissection. No signs of contrast extravasation were observed using MSCTA (Figure 1).

Due to the presenting symptoms and described dissection, the patient underwent emergency surgery under general endotracheal anesthesia on the same day. Aneurysmectomy and interposition of a Dacron tube graft (VUP MEDICA) were performed. Intimal flap fixation with Prolene 5-0 sutures and fusion of the iliac bifurcation with Prolene 3-0 sutures were performed due to the dissection of both iliac arteries. Intraoperatively, thickening of the aneurysmal sac wall with enlarged para-aortic lymph nodes was noted, and turbid content was observed during the aneurysmectomy, indicating inflammation. A swab from the operative site was obtained for culture. Following an uneventful surgical course, the patient was transferred to the Intensive Care Unit, where he received dual antibiotic therapy: meropenem 1 g IV q8h and metronidazole 500 mg IV q8h for 11 days, the choice of agent based on local institutional guidelines.

Upon admission to the microbiology laboratory of the Institute of Public Health of Vojvodina, the sample was cultured on appropriate media according to the recommended laboratory protocol [17]. After 48 h, growth was observed on anaerobic media, while the aerobic media remained sterile. Based on the characteristic morphology of the microorganisms observed on Gram-stained smears from the culture, the presence of *Campylobacter* spp. was suspected. Colonies on Shaedler agar (HiMedia, India), an anaerobic culture medium, exhibited typical characteristics of the *Campylobacter* species: small, gray, smooth, and shiny. The isolate was catalase- and oxidase-positive in terms of biochemical properties. The final identification was performed using an automated system via matrix-assisted description/ionization time-of-flight mass spectrometry MALDI-TOF (Bruker Daltonics, Billerica, MA, USA). The MALDI-TOF profile indicated *C. fetus* with a score > 2, suggesting a high probability of accurate identification. Subculturing was performed on blood agar (Oxoid, UK) and Shaedler agar under microaerophilic (5% O_2_, 10% CO_2_ and 85% N_2_) and anaerobic conditions. According to the protocol for identification of the *Campylobacter* spp. [16], a 48 h incubation at 37 °C and 42 °C was performed. Growth was observed at both temperatures. Although the literature suggest that this pathogen only grows at 37 °C and 25 °C, there are studies indicating that a certain percentage of *C. fetus* isolates can grow at 42 °C, which was confirmed in this case [18]. However, it has not been verified whether this thermotolerant strain also grows at lower temperatures. Given that the isolate was not cultured at 25 °C, we cannot conclusively state whether growth would occur at this lower temperature. Woo et al. detail that *C. fetus* growth was detected at both 25 °C and 42 °C, but, at the lower temperature, the growth of the thermotolerant strain was poor, complicating phenotypic identification at the species level [19].

Confirmation of the pathogen was also achieved by molecular methods. *C. fetus* was detected by PCR using the QIAamp DNA kit (Qiagen, Hilden, Germany) [20].

Following the initiation of empirical antibiotic therapy, blood cultures and stool samples were collected for bacterial examination. No presence of the pathogen was detected in these samples. Antimicrobial susceptibility testing was performed using the E-test gradient method (Liofilchem, Roseto degli Abruzzi, Italy), yielding minimal inhibitory concentration (MIC) values for the following antibiotics: meropenem 0.047 mg/L and imipenem 0.016 mg/L. A recommendation was made to continue therapy with meropenem for two weeks, followed by oral amoxicillin-clavulanic acid for an additional two weeks [21,22,23].

## 3. Discussion

The genus *Campylobacter* belongs to the family *Campylobacteraceae*, order *Campylobacterales*, class *Epsilonproteobacteria*, and phylum *Proteobacteria* [24,25]. Members of this genus are Gram-negative, motile, microaerophilic, non-spore-forming, oxidase-positive, rod-shaped, or curved bacteria [16,24,25]. In humans, *C. jejuni* and *C. coli* commonly cause diarrheal diseases. Additionally, members of this genus are associated with various gastrointestinal conditions such as inflammatory bowel disease (IBD), Barrett’s esophagus, functional gastrointestinal disorders, and colorectal cancer [24]. Unlike the aforementioned species, *C. fetus* rarely causes diarrhea or enteritis, but it is a significant cause of bacteremia and infections of the vascular endothelium associated with endocarditis and mycotic aneurysms [26].

Mycotic aortic aneurysms represent life-threatening conditions due to the risk of rapid aneurysm expansion and subsequent rupture, and their clinical presentation varies depending on the aneurysm location and expansion rapidity, ranging from mild to severe and rapidly progressive, thereby increasing the risk of mortality [14]. The term “mycotic aneurysm” was first introduced by Osler to describe an aortic aneurysm associated with bacterial endocarditis and the presence of vegetation on the aorta, which he described as “fresh fungus vegetation” [27]. However, the most common causative agents of such conditions are bacteria. Miller and colleagues established clinical criteria for diagnosing mycotic aneurysms, including positive germ cultures of the aortic wall and clinical signs or negative germ cultures with signs of AAA rupture [9,22]. The risk of rupture in mycotic aneurysms is reported to be between 50 and 80%, with mortality reaching up to 75% when rupture occurs [9,28]. The prevalence of mycotic aortic aneurysms in Western countries ranges from 0.6 to 2.6%, compared to 13% in Eastern countries [14].

We report a rare case of the presence of *C. fetus* in the swab of an abdominal aortic aneurysm wall. It is well known that *C. fetus* exhibits tropism toward the vascular endothelium, particularly towards damaged vascular endothelium, as well as prosthetic materials, as demonstrated by Dobrović and colleagues [26]. *C. fetus* possesses a proteinaceous capsular-like structure (S-layer) that mediates evasion of the immune response by preventing binding of the C3b complement component to the bacterial cell surface, thereby inhibiting phagocytosis and enabling the dissemination of this pathogen outside the gastrointestinal tract [29]. Vascular tropism is explained by the presence of a specific receptor on the bacterial cell surface that binds to the endothelium with a high affinity, as well as the production of local procoagulants that promote thrombus formation [30]. Consequently, this can lead to the development of endocarditis, thrombophlebitis, and mycotic aneurysms [22,29].

Infections with *C. fetus* are uncommon in humans. The primary reservoirs are the digestive and genital tracts of livestock, primarily cattle and sheep [5,19]. Infection most commonly occurs through the consumption of undercooked meat, unpasteurized milk, or direct contact with infected animals [31]. The presence alone of innate mechanisms in this pathogen to evade the immune response is not sufficient for infection in humans, but the presence of this mechanism associated with the host’s immunodeficiency can lead to the manifestation of infection caused by this microorganism. Therefore, the predisposing factors for *C. fetus* infection include various immunosuppressive conditions and diseases (e.g., HIV infection, hematological malignancies, splenectomy, cirrhosis and liver disease, and chronic alcohol consumption), cardiovascular diseases associated with altered heart valves, and the presence of prosthetic materials and pseudoaneurysms [8,32]. In our patient’s case, no immunodeficient conditions were identified except for long-term alcohol consumption, but the presence of an abdominal aortic aneurysm alone could have been a predisposing condition for the retention of this pathogen on the altered endothelium due to signifying vascular tropism. The patient denied contact with animals, as well as consumption of undercooked meat and unpasteurized milk, so the source of infection was not identified in this case.

*C. fetus* most commonly causes bacteremia, as confirmed by authors from Croatia, France, the United Kingdom, and Japan [32,33,34,35]. Bacteremia caused by this microorganism can be accompanied by endocarditis, meningitis, and mycotic aneurysms at various locations, as confirmed by the aforementioned authors. Case reports from Japan, the USA, and France describe the presence of mycotic abdominal aortic aneurysms (AAAs) in elderly patients, with the presence of atherosclerotic plaques on the aortic wall identified as a predisposing factor for the development of this urgent condition [9,22,23]. Unlike the previously mentioned cases, the presence of atherosclerotic plaques in the aortic wall was not confirmed in our patient. Unlike other species within the genus *Campylobacter*, this pathogen is rarely isolated from stool. In our case, stool cultures were negative. Blood cultures were collected only after identifying the pathogen on the abdominal aortic aneurysm wall, and they were also negative, which was expected due to the previously initiated antibiotic therapy and the time elapsed between the patient’s admission to the hospital and blood culture collection.

There are no clear recommendations for the use and duration of antimicrobial therapy for mycotic AAAs. In our patient, due to clear signs of an inflamed aneurysm with dissection signs and enlarged para-aortic lymph nodes, as well as turbid content during the aneurysmectomy, initial dual antimicrobial therapy with meropenem 1 g IV q8h and metronidazole 500 mg IV q8h was applied. The antimicrobial therapy was chosen in collaboration with infection disease specialists, based on clinical and laboratory parameters. There are no clear guidelines for antimicrobial susceptibility testing for *C. fetus* [35]. The EUCAST breakpoints provide recommendations for testing antibiotic susceptibility for two species within the genus *Campylobacter—C. jejuni* and *C. coli*. Testing susceptibility to antibiotics from the macrolide, tetracycline, and fluoroquinolone groups is recommended [36]. Several studies have examined the susceptibility of *C. fetus* to different antibiotics (MIC90), confirming that the lowest MIC values were recorded for imipenem, meropenem, ciprofloxacin, and gentamicin. Unlike the previously tested antibiotics, cefotaxime showed weak in vitro bactericidal activity [37,38] and was, therefore, not a preferred choice. Resistance of human isolates of *Campylobacter* spp. to ciprofloxacin in Serbia for the period 2014–2019 was significantly high (90%), while resistance to tetracyclines was 50% [39]. Although a low percentage of isolates resistant to erythromycin (<5%) were recorded, this antibiotic is not recommended in the therapy of *C. fetus* bloodstream infections due to described cases of treatment failure [13]. Susceptibility testing of our patient’s sample was performed using the gradient test, and the MIC values were interpreted according to PK-PD (non-species related) EUCAST breakpoints [36]. Current guidelines suggest that patients who have undergone mycotic abdominal aneurysm repair should be considered for an individualized postoperative antibiotic regimen and surveillance strategy, based on patient factors, microbiology, and the surgical technique used [10]. Carbapenems are the most common recommendation for first-line therapy for *C. fetus* infections or vascular infections [8,13]. Prolonged parenteral administration of this group of antibiotics for at least 3–4 weeks is recommended in the case of vascular infections [8]. A recent multicenter study in France indicated no resistance of *C. fetus* isolates to amoxicillin-clavulanic acid and imipenem [23]. In our case, based on the obtained low MIC values for imipenem and meropenem, the recommendation was to continue therapy with meropenem. Upon discharge, continuation of therapy with amoxicillin-clavulanic acid for the following two weeks was recommended, which was sufficient for the patient’s complete recovery. Additionally, one month after discharge, a follow-up ultrasound examination and a laboratory parameter check were performed, with results within the reference ranges. Furthermore, during the annual follow-up examination, the patient reported feeling well, with no signs of residual disease or a relapse, the abdomen was soft and painless, the scar was neat, and the femoral pulses were normal. The next annual check-up with ultrasonography of the abdomen is scheduled for November 2024.

## 4. Conclusions

We experienced a rare case of a patient with *C. fetus* infection associated with mycotic AAA. This case should highlight the importance of using modern diagnostic methods, as the ultimate outcome of such infections can be fatal. Establishing the etiological diagnosis of mycotic AAAs is crucial for prompt and adequate antibiotic therapy. Due to the lack of clear guidelines for antibiotic susceptibility testing and therapy duration, there is a need for increased surveillance of this pathogen in both human and veterinary medicine.

## Data Availability

The data presented in this study are available upon request from the corresponding author.
